# The Oxidative Cleavage of 9,10‐Dihydroxystearic Triglyceride with Oxygen and Cu Oxide‐based Heterogeneous Catalysts

**DOI:** 10.1002/cssc.202100322

**Published:** 2021-05-04

**Authors:** Andrea Vassoi, Tommaso Tabanelli, Annalisa Sacchetti, Francesca Di Gioia, Luigi Capuzzi, Fabrizio Cavani

**Affiliations:** ^1^ Dipartimento di Chimica Industriale “Toso Montanari” Alma Mater Studiorum Università di Bologna Viale del Risorgimento, 4 40136 Bologna Italy; ^2^ NovamontSpA Via Fauser 8 28100 Novara Italy

**Keywords:** azelaic acid, biomass, heterogeneous catalysis, oxidative cleavage, pelargonic acid

## Abstract

This paper deals with a new heterogeneous catalyst for the second step in the two‐step oxidative cleavage of unsaturated fatty acids triglycerides derived from vegetable oil, a reaction aimed at the synthesis of azelaic and pelargonic acids. The former compound is a bio‐monomer for the synthesis of polyesters; the latter, after esterification, is used in cosmetics and agrochemicals. The reaction studied offers an alternative to the currently used ozonization process, which has severe drawbacks in terms of safety and energy consumption. The cleavage was carried out with oxygen, starting from the glycol (dihydroxystearic acid triglyceride), the latter obtained by the dihydroxylation of oleic acid triglyceride. The catalysts used were based on Cu^2+^, in the form of either an alumina‐supported oxide or a mixed, spinel‐type oxide. The CuO/Al_2_O_3_ catalyst could be recovered, regenerated, and recycled, yielding promising results for further industrial exploitation.

## Graphical abstract

**Triglyceride cleavage**: Two copper‐based catalysts are employed for the selective oxidative cleavage of unsaturated fatty acids triglycerides to obtain pelargonic and azelaic acid, valuable products for bio‐based polymers and agrochemicals. Using oxygen as safer oxidant instead of ozone, promising results are obtained, both in terms of conversion and yields. A regeneration procedure is applied for the CuO/Al_2_O_3_ catalyst, allowing its successfully reutilization.

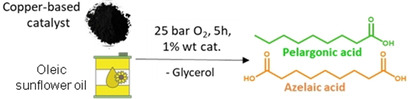

## Introduction

Oleochemistry has always been strongly linked to the concept of biorefinery, which employs vegetable oils and animal fat residues as starting materials for the synthesis of chemicals and fuels. Their similarity to petroleum compounds^[1]^ makes them suitable for transformation into marketable products and energy vectors. Moreover, they are abundantly present in nature and characterized by the presence of multiple sites for their chemical modification. Oil production and uses have been rapidly growing: since 1985 the production of oilseed (including cottonseed, sunflower oils, soybean, rapeseed, sesame seed, etc.) has increased from 190 M tons to more than 450 M tons in 2011, and nowadays it is close to 600 M tons.^[2]^ This sharp increase has been accompanied by the use of oils in a wide range of applications including fuels (bio and green diesel), lubricants, food additives, surfactants and detergents.[^1^, ^3^, ^4^, ^5^, ^6^, ^7^, ^8^] Even more importantly, they are used as monomers for the synthesis of a new generation of bio‐polymers, such as polyesters and polyamides.[^1^, ^6^, ^9^]

In this context, azelaic acid (nonanedioic acid, AA) plays a key role as a bio‐based building block. AA is used in cosmetics and pharmaceutical formulations, but also shows great potential as a plasticizer and a monomer for the synthesis of new biopolymers. A significant example is a family of polyesters based on AA and 1,4‐butanediol, both monomers being obtained from renewable sources; moreover, these polymers are also fully biodegradable and compostable.

Worthy of note is the fact that the commercial process for the synthesis of AA is accompanied by the formation of pelargonic acid (nonanoic acid, PA), which is characterized by a good antimicrobial activity and is used as sanitizing agent for food and in personal care products, as well as in the production of herbicides.^[10]^ Even more it is employed as a precursor of solvent for varnishes.^[11]^

There are currently two main industrial technologies for the synthesis of these platform molecules:

- the one‐pot cleavage (direct oxidative cleavage) of the acid (or of the corresponding triglyceride: triolein);
- the two‐step route, which includes the dihydroxylation of the double bond to the corresponding dihydroxy fatty oleic acids (or esters) and the consecutive oxidative cleavage of the diol.

Direct oxidative cleavage is the main strategy applied at an industrial scale: it is a relatively well‐developed process characterized by simplicity and good selectivity toward the target products. Indeed, it was initially patented in 1957 by Goebel et al., who were able to achieve a 78 % AA yield from the early stage of development.^[12]^ However, it uses ozone as the oxidant.^[13]^ Although the use of ozone makes it possible to prevent the formation of the stoichiometric wastes that are originated by other types of oxidants, its use entails several hazards and environmental issues, thus making it an undesirable solution for the long term.

**Scheme 1:**
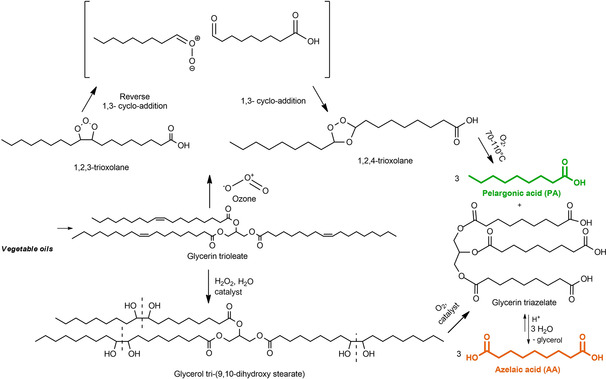
Schematic representation of the two different routes for AA and PA synthesis. At the top, the one‐pot process with ozone. At the bottom, the two‐step route with the initial formation of glycerol tri‐(9,10‐dihydroxy stearate) [Gly(DHS)] and subsequent cleavage.

The reaction steps in ozonolysis are shown in Scheme 1, in comparison with an alternative, ozone‐free mechanism. In the mechanism at the top, after a series of molecule rearrangements, ozonide is cleaved to carboxylic acids by oxidation with O_2_ at 70–110 °C without any catalyst.^[13]^ Significantly, V_2_O_5_ nanorods were reported to catalyze the entire process more efficiently.^[14]^

Some recent papers report on improvements in the ozonolysis process.[^15^, ^16^] For instance, supercritical CO_2_ could be used as a reaction solvent with ozone or KMnO_4_ as oxidants,^[16]^ making product separation possible and thus reducing the need for other energy‐intensive separations, such as distillation.

However, the needs for proper alternatives for a sustainable production of both AA and PA have prompted both academic and industrial research to seek alternative oxidants and catalytic systems. Alternative oxidants such as nitric acid, KMnO_4_, and sodium periodate[^17^, ^18^, ^19^] have been investigated, but considering their stoichiometric use, they have not proved convenient for industrial applications.

Other solutions have been considered: RuCl_3_ proved to be active not only for the direct oxidation of oleic acid with hypochlorite^[19]^ but also for the direct oxidative cleavage of oleic acid in H_2_O/MeCN/AcOEt solvent and with Na periodate oxidant,^[18]^ with ultrasonic irradiation and Aliquat 336 as a phase‐transfer agent.[^20^, ^21^] Greener oxidants such as H_2_O_2_ have also been used in combination with Os, Mo, W, and Ru catalysts.[^17^, ^22^, ^23^, ^24^] In this context, tungstic oxide supported on silica proved to be the most suitable catalyst^[25]^ for cleavage with H_2_O_2_.

In 2015, Do and co‐workers^[26]^ and Benessere et al.^[27]^ focused their research on the direct cleavage of oleic acid with H_2_O_2_ and tungstate catalyst. Metal‐exchanged zeolites (CrMCM‐41, MnMCM‐41, CoMFI, MnMFI, to name a few) were also considered in combination with supercritical CO_2_. Lastly, homogenous catalytic systems, especially Fe (OTf)_2_(mix‐bpbp) (OTf=triflouromethanesulfonate, bpbp=N,N′‐bis(2‐pyridylmethyl)‐2,2′‐bipyrrolidine), have been investigated with H_2_O_2_ and Na_2_O_2_ for the selective production of the corresponding aldehydes, while avoiding over‐oxidation to the acids.^[28]^

An alternative to the one‐pot reaction is the so‐called two‐step route, as shown in the bottom part of Scheme 1: oleic acid, or its esters (triglycerides), are first di‐hydroxylated to glycerol tri‐(9,10‐dihydroxy stearate) [Gly(DHS)], and the latter is then cleaved with O_2_,[^29^, ^30^, ^31^, ^32^, ^33^, ^34^, ^35^] with the consequent formation of PA and AA.

Santacesaria et al. studied both homogeneous and heterogeneous systems for the two‐step oxidative cleavage of oleic acid. The first step is the reaction between the fatty acid and hydrogen peroxide, with tungstic acid as the catalyst. The intermediate dihydroxystearic acid (DSA)is then cleaved with O_2_ and a Co acetate catalyst; the latter generates a polyoxometalate in‐situ by reacting with the tungstate used in the first step.^[36]^ Hydrogen peroxide was employed in other studies for the cleavage of oleic acid and its ester by.[^37^, ^38^, ^39^]

Other metals and oxidants were used, such as supported nickel and formic acid combined with H_2_O_2_ by Lemaire et al.,^[40]^ or tungstic acid followed by cleavage with sodium hypochlorite.^[41]^ Kulik et al.[^29^, ^30^] reported the oxidative cleavage of diols from oleic acid using supported Au/Al_2_O_3_ and O_2_ as the oxidant, obtaining AA and PA in yields of 86 and 99 %, respectively. The catalytic activity, however, significantly decreased after repeated uses. As a matter of fact, after the first recycle, the conversion declined from 94 to 77 %, while the yields of AA and PA decreased by approximately 30 %. Moreover, a large amount of NaOH was necessary to activate the substrate. Lastly, a recent work by Melchiorre et al.^[42]^ reports the use of diperoxo‐tungsten complex as a homogeneous catalyst for the synthesis of PA and AA starting from oleic acid.

Starting from these previous studies and experiences, we wanted to optimize a heterogeneous catalyst used for the selective cleavage of the Gly(DHS), in accordance with green chemistry principles. First of all, no solvents were used during the reaction: the starting material, originally a thick yellow grease, was melted and introduced into the reactor. Secondly, we wanted to avoid the use of metals such as Co or Cr^[43]^ in catalyst formulation, because their toxicity would lead to health issues if they leached into the reaction medium. Therefore, after an initial screening of different catalytic systems, we studied Cu^2+^ in two different forms:

- CuO supported on alumina.
- Cu in a spinel‐type ferrite system (Cu_0,6_Fe_2,4_O_4,2_ with Cu/Fe atomic ratio of 1 : 4).

Lastly, we demonstrated the stability of CuO/Al_2_O_3_ after calcinations for different reaction cycles.

## Results and Discussion

### Preliminary screening of catalysts

The detailed characterization of the catalytic materials investigated is described in the Supporting Information (Figures S1–S5 and S9, Tables S1 and S2).

Preliminary screening tests were conducted as detailed in the Experimental Section, while Table 1 summarizes the results obtained, compared with a benchmark catalyst made of Co and tungstate anion.[^32^, ^33^, ^34^, ^44^]

**Table 1:** Results of reactivity experiments with heterogeneous catalysts, preliminary screening, and comparison with the benchmark homogeneous catalyst.^[a]^

Catalyst	Gly(DHS) conversion [%]	PA yield [%]	AA yield [%]
no catalyst	24±2	8±1	16±2
Au/TiO_2_	43±3	12±1	16±2
H_4_[PW_11_Fe (H_2_O)O_39_]	50±3	7±1	11±1
Fe_3_O_4_	20±2	8±1	11±1
CoFe_2_O_4_	27±2	17±1	22±2
NiFe_2_O_4_	27±2	25±2	26±2
CuFe_2_O_4_	96±3	52±2	70±2
CuO/Al_2_O_3_	87±3	62±2	76±2
benchmark: Co^2+^/WO_4_^2−^	93±3	31±2	34±2

[a] Reaction conditions: 1 wt % catalyst; *T*: 80 °C; stirring rate: 500 rpm; *P* O_2_: 25 bar; reaction time: 5 h. Note: AA was present in the form of glycerol triazelate Gly(TA); it underwent methanolysis during the derivatization for GC analysis, and was analyzed as methyl ester. Yields and conversion have been calculated as explained in the Experimental Section.

Both Au/TiO_2_ and polyoxometalate showed a relatively high Gly(DHS) conversion, but with yields to AA and PA that were similar to or lower than those achieved with no catalyst at all; this means that, under the conditions examined, the oxidative scission of glycol mainly led to products other than AA and PA. Regarding the Au/TiO_2_ catalyst, it is worth noting that we conducted the reaction without any solvent and no addition of base: conditions that are different from those reported in literature for this catalyst when used for the oxidative scission.^[30]^

Co and Ni spinels showed remarkably lower activity compared to the reference homogeneous catalyst; however, yields to both PA and AA were higher than those recorded with no catalyst, thus suggesting that these materials can transform glycol with good selectivity. The situation was different in the case of the Cu spinel, which showed high glycol conversion and excellent yields to both PA and AA.

The same experiment was conducted with Cu spinel and adding Na_2_WO_4_/H_2_O. Significantly, the resulting yields to PA and AA in this case were equal to 13 and 20 %, like those obtained with the reference catalyst, but lower than those obtained in the absence of tungstate.

Catalysts based on CuO supported on alumina gave good catalytic results, with high glycol conversion and yields to both acids close to those shown by the reference catalyst.

After this initial screening of a wide range of catalysts, we decided to further investigate the catalytic performance of CuO/Al_2_O_3_ and CuFe_2_O_4_, which showed quite promising reactivity patterns.

### Catalysts based on commercial CuO/Al_2_O_3_

**Figure 1:**
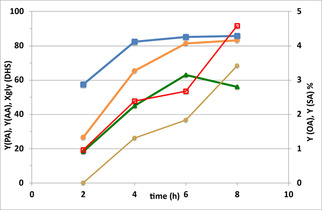
Catalytic performance as a function of reaction time. Catalyst CuO/Al_2_O_3_. Symbols: Gly(DHS) conversion (▪), yields of AA (•), PA (▴), OA (**○**), SA (□). OA and SA are on the secondary Y scale. Temperature 80 °C.

Figure 1 shows the catalytic performance according to time for the commercial catalyst made of CuO over Al_2_O_3_ (surface area 171±4 m^2^ g^−1^, pore volume 0.37 cm^3^ g^−1^, average pore diameter 8.5 nm, Figure S4). Cu content was determined by energy‐dispersive X‐ray spectroscopy (EDX) resulting in 14.1 wt % (17.6 wt % CuO, Figure S8).

Results show that at 25 bar O_2_ pressure and a temperature of 80 °C Gly(DHS) conversion as high as 57 % was reached after a reaction time of just 2 h, rising to 80 % after 4 h; longer reaction times led to a slower increase of conversion. AA and PA yields showed a similar increase, confirming that they formed at the same time; however, their yield was very low after 2 h and rapidly increased with longer reaction times, suggesting that they formed via a consecutive reaction network, by the transformation of an intermediate compound.

As detailed in the Experimental Section, the starting glycol reactant already contained by‐products, originating from the first dihydroxylation step. Among them, nonanal and 9‐oxononanoic acid were converted during the second step until their concentration became close to 0.3 wt %. This might suggest that these two aldehydes were intermediates for AA and PA production. In particular, nonanal leads to PA formation and 9‐oxononanoic acid to AA.

Conversely, the amount of octanoic acid and suberic acid (the C_8_ counterparts of PA and AA) showed an almost linear increase over time, with almost identical trends; this indicates that they did not form by consecutive transformations of C_9_ aldehydes or C_9_ acids. As highlighted in ref. [23], shorter‐chain by‐products should be formed if the reaction pathway includes an over‐oxidation or oxidative degradation, following a radical reaction pathway. The formation of products with only C_8_ atoms might be explained with keto‐containing intermediates such as 9(10)‐hydroxy‐10(9)‐oxostearic acid (hydroxyketone, HK) and 9,10‐dioxostearic acid (diketone, DK).^[30]^

Palmitic acid (saturated C_16_ monocarboxylic acid) and stearic acid (saturated C_18_ monocarboxylic acid) were also present in the reaction mixture and their content decreased slightly during the reaction. Palmitic acid decreased from the initial 3.9 down to 3.5±0.2 wt %, while stearic acid fell from 2.5 to 2.0±0.2 wt %.

Other by‐products formed during the oxidative cleavage step were valeric acid (pentanoic acid), hexanoic, and heptanoic acid, with a maximum yield after an 8 h reaction time equal to 0.2, 1.0, and 0.3 %, respectively (values not shown in Figure 1).

Figure S6 shows that the reaction mixture was quite clear and pale yellow after a 4 h reaction, while as the reaction time increased the yellow colour became browner and more and more intense after a reaction time of 8 h. This phenomenon could be attributed to the over oxidation of PA and AA.

**Figure 2:**
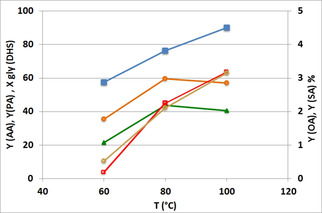
Catalytic performance as a function of temperature. Catalyst CuO/Al_2_O_3_. Symbols: conversion (▪), yields of AA (•), PA (▴), OA (**○**), SA (□). OA and SA are on the secondary Y scale. Reaction time 5 h. Other conditions as in Table 1.

The effect of temperature is shown in Figure 2. The optimal temperature range was 80–100 °C; there not only were the PA and AA yields were the highest, but the reaction mixture was also clear and free from heavier (dark) by‐products (see Figure S7).

Reusability tests were conducted: at the end of each reaction experiment, conducted for 6 h at 80 °C, the catalyst was recovered by means of centrifugation (4500 rpm for 15 min), then washed with acetone (3 times, 15 mL acetone), and then re‐used for another experiment. The catalyst showed a poorer performance already after the first use, with a decline in glycol conversion (24 %) and yield to AA (15 %) and PA (12 %). Indeed, the thermogravimetric analysis (TGA) of the catalyst after its first use (and after washing with acetone) showed an 18 % weight loss at 600 °C in air, whereas the fresh catalyst showed only an 8 % weight loss at the same temperature. The IR spectra (Figure S8) of the used catalyst highlighted the presence of adsorbed organic compounds, which were not removed during washing and hence were strongly bound on the catalyst surface. For a more drastic treatment, the spent catalysts were heated in air at 500 °C, for 3 h. The activity after this treatment was recovered, with a yield to AA of 60±2 % and to PA of 57±2 % (results for the fresh catalyst are shown in Figure 2).

### Catalysts based on Cu ferrospinels

In previous papers, we investigated the reactivity and redox properties of Cu ferrospinels in the chemical‐loop reforming of ethanol.[^39^, ^45^] It was found that the reduction of Cu and Fe in CuFe_2_O_4_ follows an “autocatalytic” model, in which the primarily formed Cu^0^ nuclei activate the reducing gas (H_2_ or C_2_H_5_OH), which in turn catalyses the further reduction of the newly reformed Fe‐enriched spinel. In general, the redox properties of M‐modified ferrospinels were found to depend strongly on the nature of the incorporated cation (Co, Cu, Mn or Cu/Co, Cu/Mn, Co/Mn), and the ratio between M and Fe.

**Figure 3:**
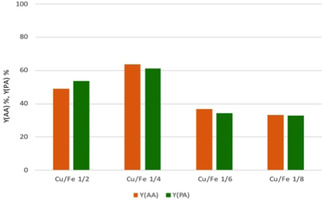
Comparison between copper ferrites calcined at 450 °C with different Cu/Fe ratio. Reaction conditions as in Table 1. Reaction time 5 h.

We first tested catalysts with different Cu/Fe ratio (Figure 3); the main chemical physical features of samples (reported in the Supporting Information) showed that for a Cu/Fe atomic ratio equal to 1/2 (corresponding to the classic stoichiometry for metal ferrites CuFe_2_O_4_), not all of the Cu was incorporated into the spinel structure, and some amount of CuO was formed. Conversely, lower contents of Cu led to a monophasic compound, with the typical features of ferrospinel.^[46]^

Because of its superior performance, we focused on the spinel with Cu/Fe atomic ratio equal to 0.25 (1 : 4). The effect of the calcination temperature was also investigated: 450 °C appeared to be the optimal annealing temperature for these spinels^[46]^ (Figure S9, Table S2*)*.

**Figure 4:**
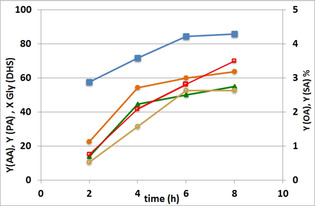
Catalytic performance as a function of reaction time. Catalyst Cu/Fe/O ferrospinel (Cu/Fe atomic ratio=1 : 4). Symbols: conversion (▪), yields of AA (•), PA (▴), octanoic acid OA (**○**), suberic acid SA (□). OA and SA are on the secondary Y scale. Temperature 80 °C.

**Figure 5:**
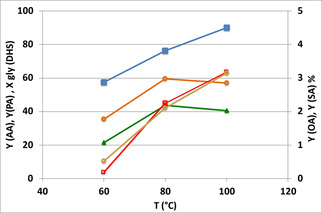
Catalytic performance as a function of temperature. Catalyst Cu/Fe/O ferrospinel (Cu/Fe atomic ratio=1 : 4). Symbols: conversion (▪), yields of AA (•), PA (▴), octanoic acid OA (**○**), suberic acid SA (□). OA and SA are on the secondary Y scale. Reaction time 5 h. Other conditions as in Table 1.

Figures 4 and 5 show the effect of reaction time and temperature on the catalytic performance. The spinel behavior was similar to that shown by CuO/Al_2_O_3_; in this case, some of the compounds present in the starting reactant mixture (i. e., nonanal and azelaic aldehyde) were also oxidized and no longer present in the reaction mixture after the oxidative cleavage. The amount of palmitic acid and stearic acid was reduced, and the final yield was between 1.5 and 2 % for both compounds; yields to octanoic acid and suberic acid were no higher than 1.0 and 1.8 %, respectively.

Nevertheless, the spinel catalyst turned out to be less active than that based on supported Cu oxide (with similar value of the surface area), as shown by the performance comparison (Figures 1 and 4). In this case, the maximum temperature applied was 100 °C, to limit any effect of metal species dissolution.

**Figure 6:**
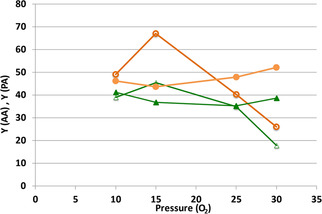
Catalytic performance as a function of O_2_ pressure. Catalysts CuO/Al_2_O_3_ (full symbols) and Cu/Fe/O ferrospinel (Cu/Fe 1 : 4) (open symbols). Symbols: yield of AA (•,**○**) and yield of PA (▴, **Δ**). Temperature 80 °C; reaction time 3 h; catalyst amount 1 wt %.

However, one main difference between the two catalysts is shown in Figure 6, where the effect of O_2_ pressure on the catalytic behavior (3 h reaction time) is compared.

It appears that the performance of the catalyst based on supported CuO was not affected by O_2_ partial pressure; this means that the activation of molecular oxygen on the Cu active species is not the rate‐limiting step. The situation with the catalyst based on Cu ferrite was quite different. With this catalyst, in the low O_2_ pressure range, yields to both AA and PA were proportional to oxygen pressure; this suggests that O_2_ plays a role in the generation of active species, the latter event being the rate‐determining step. For oxygen pressure higher than 15 atm, however, a steep decline in the yield to both AA and PA was seen, due to the formation of oxidative degradation compounds. Indeed, the catalytic behavior under a 15 atm oxygen pressure was considerably better than that seen under a 25 atm oxygen pressure, which was used in Figures 4 and 5.

**Figure 7:**
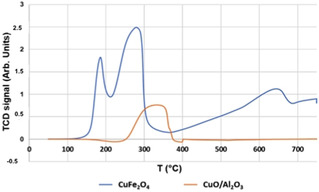
TPR profile for CuO/Al_2_O_3_ (orange line) and CuFe_2_O_4_ (blue line).

The different performance shown by the two catalysts was due to their different redox properties, as shown in Figure 7, where the temperature‐programmed reduction (TPR) profiles of samples are reported.

In the case of the CuO/Al_2_O_3_ catalyst, the reduction signal of Cu^2+^ starts at 250 °C with a maximum between 320 and 340 °C. In the case of the ferrospinel, the reduction profile shows a peak at a *T* lower than 200 °C and a major peak with maximum rate between 250 and 300 °C; the latter is attributed to a primary stepwise reduction of the spinel with final formation of Cu^0^ and Fe_3_O_4_ phases.^[46]^ The enhanced reducibility of Cu^2+^ in the spinel may correspond to a more difficult reoxidizability of the reduced Cu species, which in turn may explain its limited ability to activate molecular oxygen. On the other hand, it seems that an excessive concentration of active oxygen species may be responsible for the decline in selectivity to AA and PA seen at high O_2_ pressure.

## Conclusion

Spinel‐type copper‐ferrite and CuO/Al_2_O_3_ catalysts were investigated for the second step in the two‐step oxidative cleavage of stearic acid, carried out with oxygen and in the absence of any solvent. The two catalysts showed their best performance under different conditions, because of their different redox properties. Molar yields of azaleic acid equal to 63 and 83 % were obtained with copper ferrite and copper oxide catalysts respectively, with corresponding yields towards pelargonic acid of 70 and 62 %. The spinel‐type catalyst showed a behavior significantly dependent on oxygen partial pressure, which was not the case for the supported Cu oxide catalyst. The latter catalyst could be recovered and reused after a calcination treatment.

## Experimental Section

### Catalyst preparation

The syntheses of the main catalysts (Cu ferrospinels and CuO/Al_2_O_3_) are described below, while the catalysts used for the screening tests are reported in the Supporting Information.

**Ferrospinels**: Catalysts were prepared using the co‐precipitation method, as reported in literature.^[47]^ The materials synthesized belong to the class M_*x*_^2+^Fe_(1−*x*)_^2+^Fe_3−(1−*x*)_^3+^O_*y*_^2−^, where M^2+^ is either Fe^2+^, Co^2+^, Ni^2+^, or Cu^2+^ (*x*=0 corresponds to the unmodified magnetite). The chemicals used for the preparation were: Fe(NO_3_)_3_ ⋅ 9H_2_O (Sigma‐Aldrich, 98 %), Cu(NO_3_)_2_ ⋅ 2.5H_2_O (Sigma‐Aldrich, 98 %), Mg(NO_3_)_2_ ⋅ 6H_2_O (Sigma‐Aldrich, 99 %), Fe(SO_4_) (Merck, 99,50 %), NaOH (Sigma‐Aldrich, >99 %). The mixed solutions of metal precursors containing 50 mL of 1 m Fe(NO_3_)_3_ and 50 mL of 0.5 m M^II^(NO_3_)_2_ (where M=Co, Ni, Mn, or Cu) were dropped into a separation funnel and then added drop‐by‐drop into the reaction vessel containing 500 mL of NaOH aqueous solution (2 m) at the temperature of 45 °C, under vigorous stirring, with continuous monitoring of the pH (kept above 13). For the synthesis of magnetite, Fe(SO_4_) ⋅ 7H_2_O was preferred as the precursor instead of nitrate salt. Finally, the suspension was digested for 2 h at 45 °C. The precipitate was recovered by vacuum filtration and washed with at least 1.5 L of demineralized water at RT, to remove both sodium and nitrate/sulfate ions. The washed samples obtained by co‐precipitation were dried at 120 °C in air for 2 h; for magnetite, the drying temperature was kept at 80 °C in order to avoid the oxidation of Fe^2+^ to Fe^3+^. These compounds were the “precursors” of the desired mixed oxides. After drying, the solids were milled in an agate mortar and then annealed in static air at 450 °C for 8 h, by using a temperature ramp of 10 °C min^−1^. As far as the magnetite is concerned, this was thermally treated in inert atmosphere by heating under a N_2_ flow at 450 °C for 8 h, to prevent the oxidation of Fe_3_O_4_ into hematite (Fe_2_O_3_).

**Supported Cu oxide catalyst**: CuO and CuO supported over alumina [CuO/Al_2_O_3_, pellets (14–20 mesh), with nominal CuO loading 13 % by weight] were purchased from Merck and Sigma‐Aldrich, respectively.

### Catalyst characterization

The specific surface area and total pore volume of the catalysts were measured using the Brunauer‐Emmett‐Teller (BET) model, by physisorption of liquid nitrogen at −196 °C using a MICROMERITICS ASAP 2020 instrument.

X‐ray diffraction (XRD) analyses were conducted in a Philips PW 1050/81goniometric diffractometer (Bragg‐Brentano geometry) with a PW 1710 chain counting. The CuK_α_ radiation, made monochromatic by means of a nickel filter with *λ* of 0.15418 nm, was used for the analysis: the acquisition region was 5°<2*θ*<80°, with steps of 0.1° and count of intensity every 2 s. The range of analysis is 20°<2*θ*<80° with a scanning rate of 0.05° s^−1^ and time‐per‐step=1 s. For the interpretation of the patterns and phase identification software from PANalytical Company and the FIZ Karlsruhe‐ICSD Database were used. The Debye–Scherrer equation was used for the calculation of crystallite dimensions, which is related to the full width at half maximum (FWHM).

The temperature‐programmed reduction‐oxidation‐reduction (TPR_1_‐O‐R_2_) analysis was performed using a MicromeriticsAutochem 2 2920 V 4.05 Chemisorption Analyzer. The sample was initially pre‐treated with an inert gas flow [gas: He; flow: 30 mL min^−1^; temperature ramp: 150 °C for 10 min (10 °C min^−1^)], then heated up under programmed controlled temperature and H_2_/O_2_ flows: (TPR_1_): gas: 5 % H_2_/Ar; flow: 30 mL min^−1^; temperature ramp: 750 °C for 30 min (10 °C min^−1^); Temperature programmed oxidation (TPO): gas: 5 % O_2_/Ar; flow: 30 mL min^−1^; temperature ramp: 750 °C for 30 min (10 °C min^−1^); Temperature programmed reduction‐2 (TPR_2_): as for TPR_1_.

The desorbed species from the materials were measured by means of a thermal conductivity detector (TCD) and a quadrupole MS.

### Catalytic tests

Initially, a catalyst screening was conducted with an autoclave system with six small independent vessels (Amar equipment Pvt. Ltd. Eco 6‐25, design pressure 100 bar). Subsequently, the best‐performing catalysts were selected and tested in a 100 mL Amar autoclave (Amar equipment Pvt. Ltd. Eco) equipped with a Teflon vessel for preventing the corrosion caused by the carboxylic acids produced during the reaction.

The starting oleic oil mixture had the following fatty acids composition: 83.8 wt % oleic acid, 7.9 wt % linoleic acid, 0.1 wt % linolenic acid, 4.2 wt % palmitic acid, 2.8 wt % stearic acid, 0.1 wt % palmitoleic acid, 0.1 wt % arachidic acid, 0.1 wt % behenic acid.

During a first dihydroxylation step, the unsaturated fatty acids tryglycerides were converted to vicinal glycols. This mixture contained 70.6 % of Gly(DHS) and more products of further oxidation; it was employed as the starting material for the oxidative cleavage reported in this paper.

During screening tests, 10 g of the starting diol was introduced into each vessel, while 15 g was fed into the large autoclave; due to the lack of solvent, Gly(DHS) had to be previously melted at roughly 50 °C. The oxidative scission of Gly(DHS) was performed at 80 °C, 25 bar, and the reaction was performed for 5 h. A previous test made it possible to define the optimal catalyst load (1 % wt referring to the starting material) and stirring rate (500 rpm). Unless stated otherwise (e. g., catalytic tests according to temperature, time, or oxidant pressure), the described conditions were used in all reactivity experiments. The apparatus was sealed, vented with nitrogen, and finally pressurized with the required gas (usually oxygen). The system was heated and stirred during the reaction; the “time zero” of the reaction was taken when the reactor reached the desired reaction temperature. At the end of the reaction time, the heating was turned off, and the autoclave was cooled down without interruption of the stirring until the temperature of 50 °C was reached. Then the system was opened, and the reaction mixture was transferred into Falcon centrifuge tubes for catalyst recovery by means of centrifugation (4500 rpm for 15 min).

GC analysis required previous derivatization of the acids: 0.1 g of the reaction mixture was dissolved in 1 mL of toluene with addition of 450 μL of internal standard (solution prepared by dissolving 1 wt % of 10‐undecenoic acid and 0.8 wt % of nonadecanoic acid in 10 mL of MeOH). Subsequently, 2.8 mL of BF_3_ in methanol (10 wt %) and 150 μL 2,2‐dimethoxypropane were added as scavenger to prevent water interferences with the derivatization procedure. The solution was heated at 80 °C for 1 h. Lastly, the obtained fatty acid methyl esters (FAMEs) were separated by CHCl_3_ and, after a drying step with anhydrous Na_2_SO_4_, the sample was ready to undergo analysis.

Analyses were performed using a Shimadzu GC‐2025AF model, equipped with an AOC‐20i auto‐injector and flame ionization detector (FID) as the detector. A polar Agilent J&W DB‐23 capillary column (stationary phase consisting of 50 %‐cyanopropyl‐methylpolysiloxane) was used for an optimal separation of FAMEs.

### Conversion and yields: expression of results

The final crude mixture is mainly made of carboxylic acids: some are present as free fatty acids, like PA and octanoic acid (OA), while AA and suberic acid (SA) are found in the form of glyceril‐tricarboxylate [Gly(TA) and Gly(SA)].

To make the quantification via GC‐analysis possible, it is necessary to perform a derivatization of the crude mixture by means of transmethylation (for details see the “Catalytic tests” section). In this procedure, both the transmethylation and methanolysis of fatty acids and triglycerides are promoted. Therefore, Gly(TA) is converted into glycerol and three equivalents of methylated AA, and the free acids are converted into the corresponding methyl esters.

Starting from a complex mixture of carboxylic acids derived from a high‐oleic sunflower oil (HOSO) after a first di‐hydroxylation step, Gly(DHS) conversion [*X*_Gly(DHS)_] was calculated considering its initial amount as 70.6 %. The formula used is the following [Eq. 1]:

(1) $$X_{Gly\left(DHS\right)}=\frac{{mol}_{Gly\left(DHS\right)}^{0}-{mol}_{Gly\left(DHS\right)}^{\;}}{{mol}_{Gly\left(DHS\right)}^{0}}\times 100$$

Yields to both the main products, PA and AA, and minor products, SA and OA, were expressed as shown in Equation 2:

(2) $$Y_{i}=\frac{\left(\frac{mol\;i}{{mol}_{Gly\left(DHS\right)}^{0}}\right)\times \left(\frac{C\;atoms\;of\;i}{C\;atom\;of\;Gly\left(DHS\right)}\right)}{Y_{i,\;max}}\times 100$$

where *i*=PA, AA, SA, or OA, and the number of C atoms of Gly(DHS)=57; number of C atoms of AA and PA=9; number of C atoms of OA and SA=8. In this way, the molar yield of each product is normalized with respect to both the C atoms ratio between the product *i* and Gly(DHS), and the maximum molar yield (*Y*_*i*,max_) obtainable for that compound.

*Y*_*i*,max_ is calculated by considering the stoichiometric coefficients, as shown in Equation 3:

(3) $$Y_{i,max}=3\times \frac{C\;\mathrm{atoms}\;\mathrm{of}\;i}{57}\times 100$$

Equation (3) was used to obtain the maximum molar yield for each compound [note: the number “3” in the formula refers to the number of moles for each product formed from 1 mol of starting Gly(DHS)].

*Y*_*i*,max_ is equal to 47 % for AA and PA and 42 % for SA and OA. In fact, under the hypothesis of a 100 % conversion of Gly(DHS) into PA and AA, the sum of the *Y*_*i*,max_ would be equal to: 47 % for PA [9×3=27 C atoms out of the starting 57 C atoms of Gly(DHS)], plus 47 % for AA (9×3=27 C atoms out of the starting 57 C atoms), plus 6 % for glycerol (3 C atoms out of the starting 57 C atoms), with an overall consumption of C atoms equal to 100 %.

The main reaction scheme was reported in the introduction (Scheme 1), but the formation of other by‐products caused by over‐oxidation was investigated, as shown in the Supporting Information (Figure S10).

## Conflict of interest

The authors declare no conflict of interest.

## Supporting information

As a service to our authors and readers, this journal provides supporting information supplied by the authors. Such materials are peer reviewed and may be re‐organized for online delivery, but are not copy‐edited or typeset. Technical support issues arising from supporting information (other than missing files) should be addressed to the authors.

Supplementary
