# Supplementary material for: The Oxidative Cleavage of 9,10‐Dihydroxystearic Triglyceride with Oxygen and Cu Oxide‐based Heterogeneous Catalysts
Source: ChemSusChem. 2021 May 4;14(11):2375–82. doi: 10.1002/cssc.202100322 (PMC8251944; doi:10.1002/cssc.202100322)
Supplement: Supplementary file 1 — Supplementary [file CSSC-14-2375-s001.pdf]

ChemSusChem

Supporting Information

The Oxidative Cleavage of 9,10-Dihydroxystearic Triglyceride with Oxygen and Cu Oxide-based Heterogeneous Catalysts

Andrea Vassoi, Tommaso Tabanelli, Annalisa Sacchetti, Francesca Di Gioia, Luigi Capuzzi, and Fabrizio Cavani*© 2021 The Authors. ChemSusChem published by Wiley-VCH GmbH. This is an open access article under the terms of the Creative Commons Attribution License, which permits use, distribution and reproduction in any medium, provided the original work is properly cited.

Characterization

A *Brucker Alpha Platinum* IR spectrometer was used for Attenuated Total Reflection (ATR)Infrared spectroscopy.IR spectra were taken with an average of 64 scan per sample at a resolution of 2 cm^{-1}.

The measurement of Dynamic Light Scattering (DLS) was done with a *Zetasizer Nanoseries* (Malvern Instruments diluting 10 drops of samples in 10 mL of water.

The X-ray fluorescence (XRF) measurements were done with a *PANalytical Axio sAndvanced* dispersive wavelength spectrometer equipped with rhodium tube of 4 kW power. The measurements were performed under vacuum. The samples were grinded and mixed with an organic wax (300 mg sample, 100 mg wax) before being pelletized.

For diffuse Reflectance UV spectroscopy the instrument used was a *Perkin Elmer Lambda 19* spectrometer, equipped with a calcium sulphate integrating sphere; the band of analysis was 190 nm <l< 450 nm.

Thermo gravimetric Analysis (TGA) were conducted in a *TA SDT Q600* instrument, loading 20 mg of sample in an Al_2O_3 – pan and heated up to 900°C with a ramp of 10°C/min while 100 mL/min of nitrogen (or air) was continuously fed.

Before TEM analysis, samples were crushed, suspended in ethanol and homogenized using ultra-sonication (for 5 min). The suspension was deposited on a holey-carbon film Cu-grid for TEM analysis, then dried at 100°C. The analyses were carried out by means of a microscope TEM/STEM FEI TECNAI F20 at 200 keV. SEM analyses were performed by using a *SEM Zeiss EP EVO 50* with a tungsten electron source and secondary and backscattered electrons detectors. The E.D.X. was an *Oxford Instruments INCA ENERGY 350* including a Si(Li) detector equipped with an ultrathin window (Z > 4 (Be)). The images were collected in a high vacuum mode at EHT = 20 keV and the spectra were registered for a 60 s collection time.

Characterization of $Cu_{0,6}Fe_{2,4}O_{4,2}$

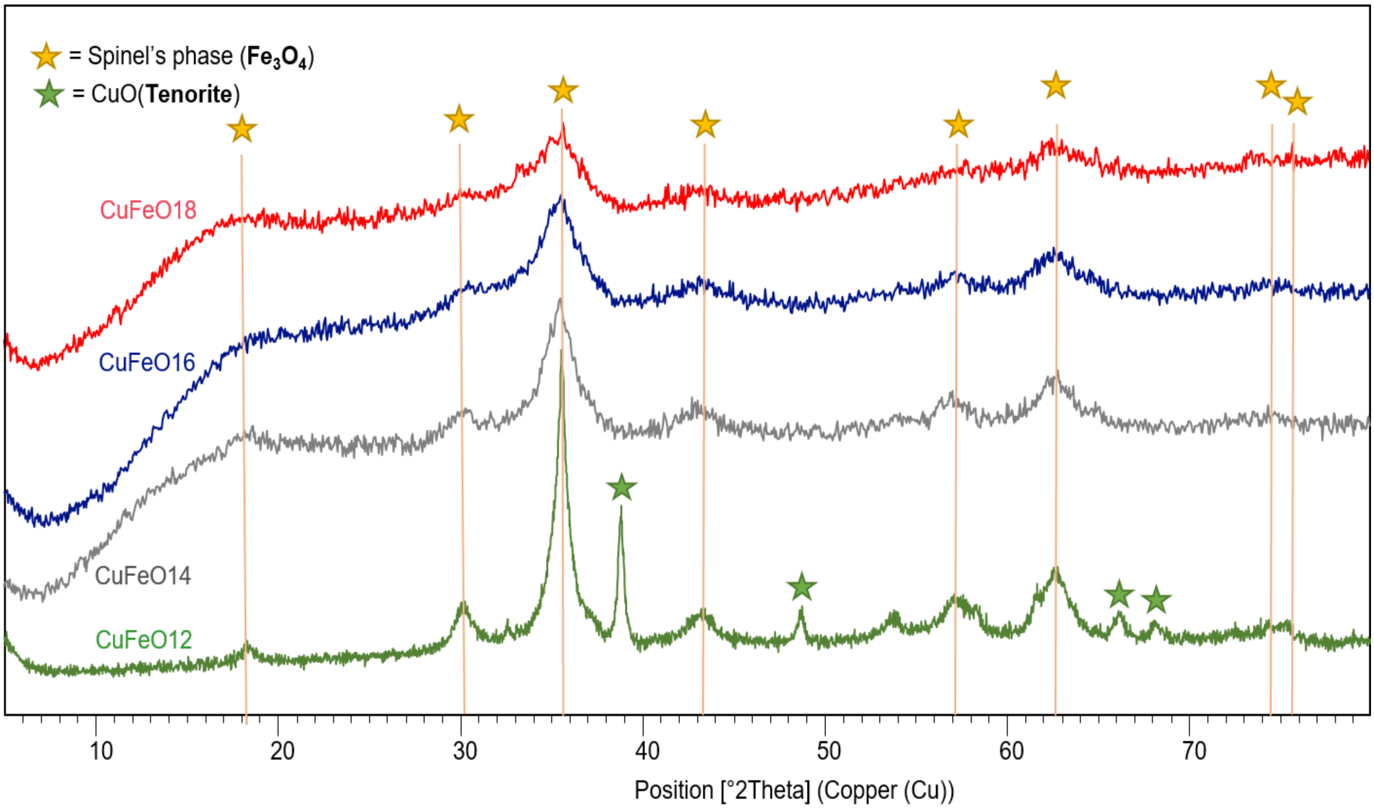

Figure 1: XRD pattern of Copper Ferrite with different Cu /Fe ratio

Table 1: determination of the surface area of the copper-ferrites catalysts determined by B.E.T. analysis.

SAMPLE	Ratio Cu/Fe	$S_{B.E.T.}$ (m^2/g)
$Cu_{0,33}Fe_{2,67}O_{4,3}$	1:8	64
$Cu_{0,43}Fe_{2,57}O_{4,2}$	1:6	98
$Cu_{0,6}Fe_{2,4}O_{4,2}$	1:4	86
$CuFe_2O_4$	1:2	67

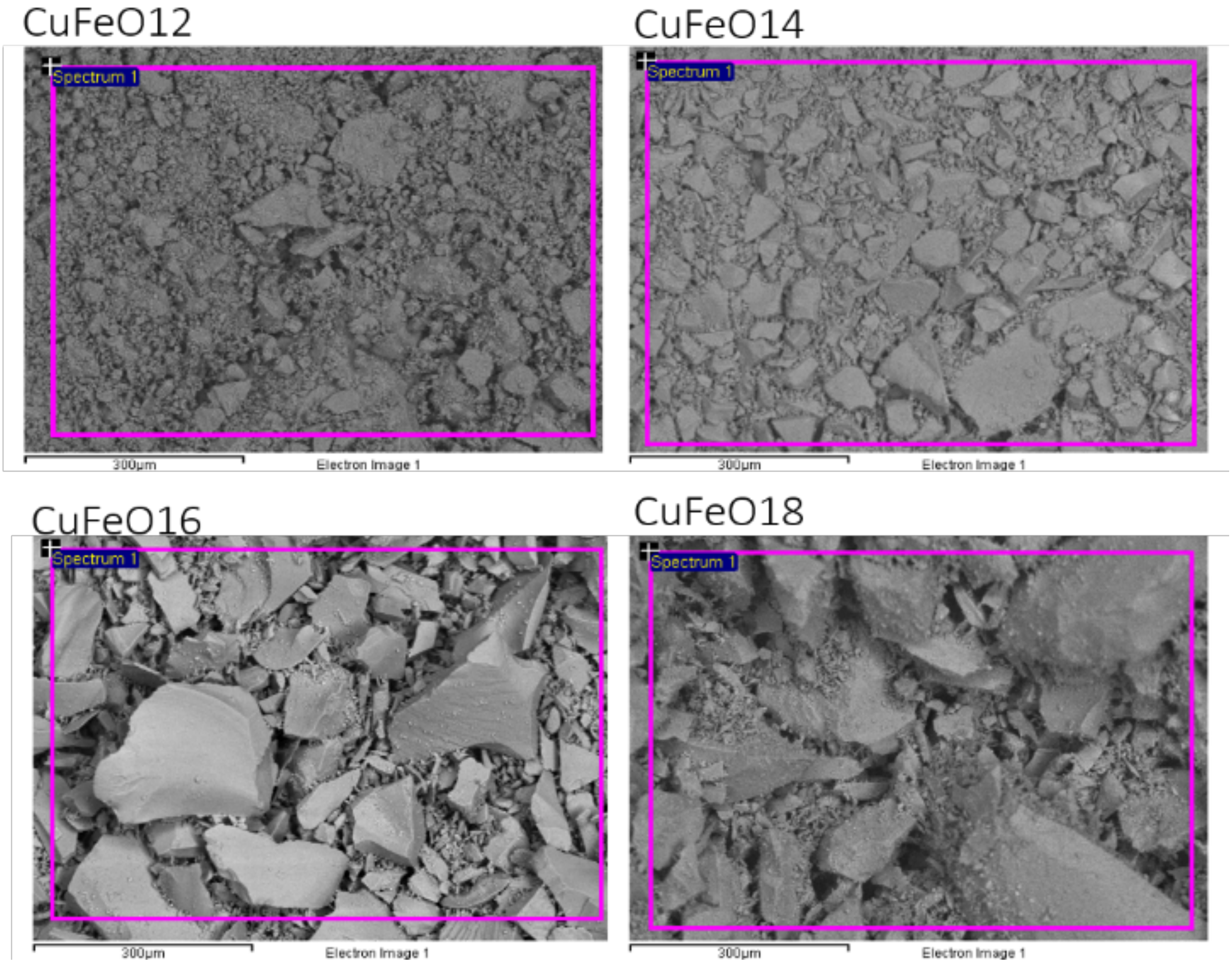

Figure 2: SEM images of copper ferrite with different Cu/Fe ratio

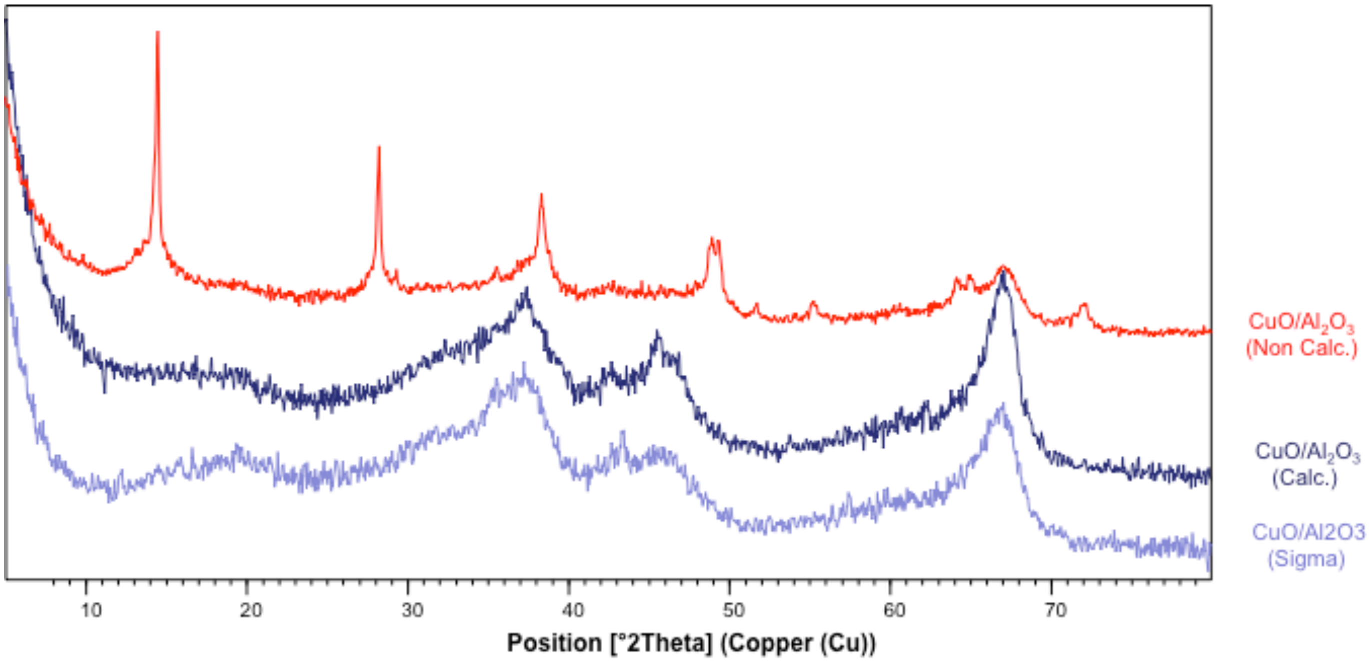

Figure 3: XRD pattern of CuO/Al2O3

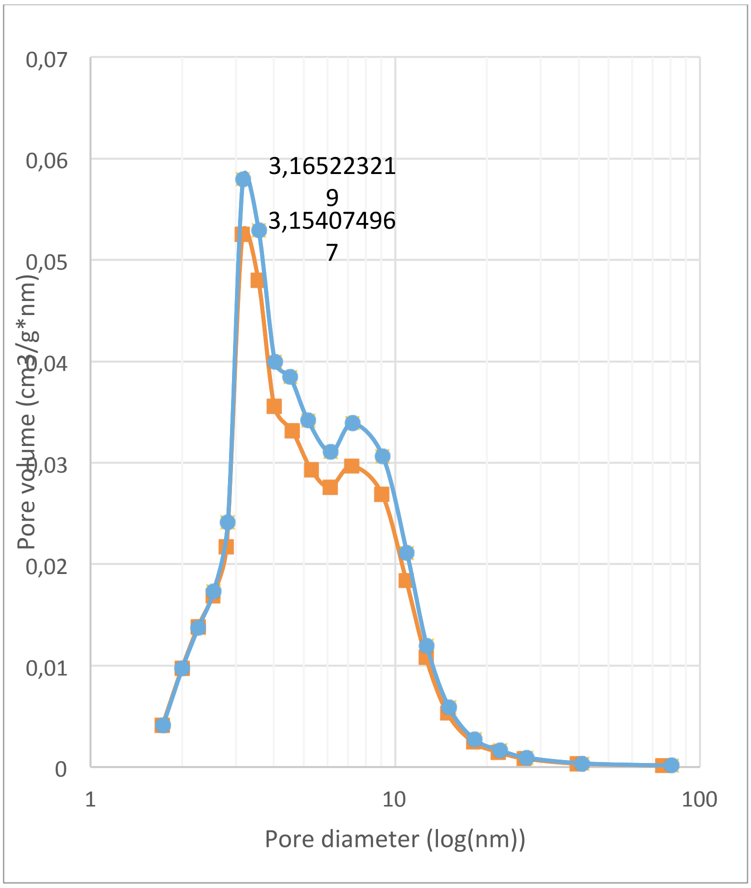

Figure 4: Pore volume analysis performed over CuO/Al_2O_3

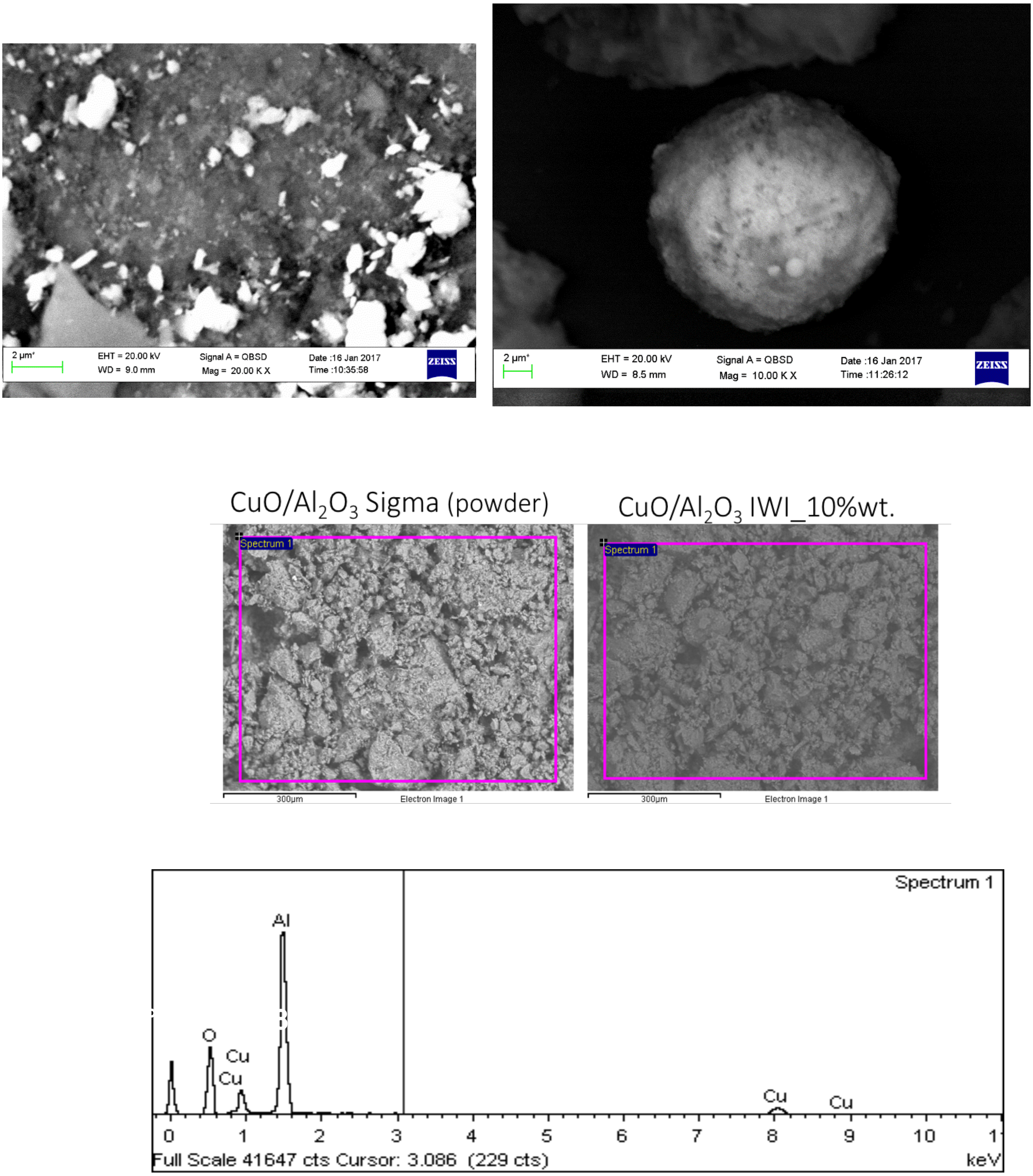

Figure 5:: TEM images of CuO/Al_2O_3, both commercial (on the left) and synthesized n the laboratory (on the right). SEM-EDX analysis of CuO/Al_2O_3

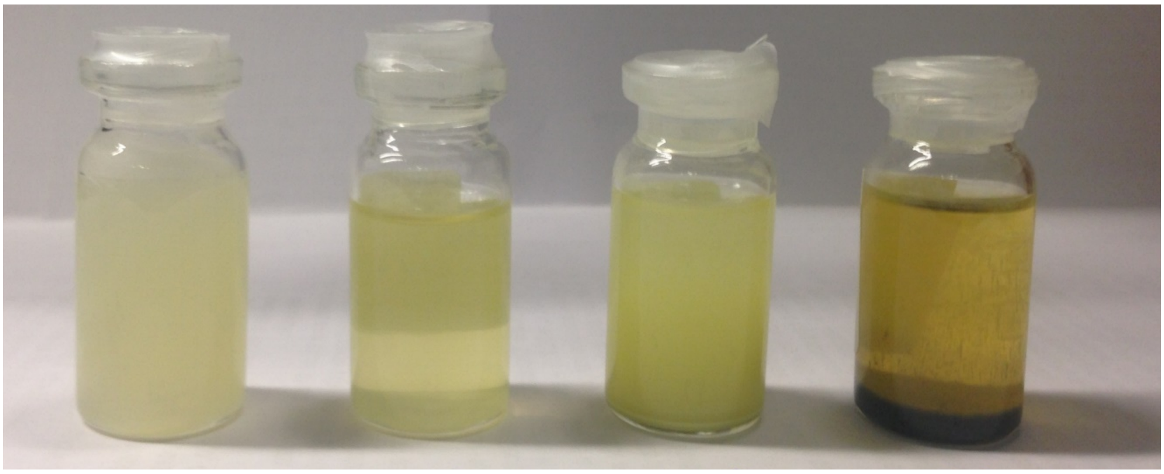

Figure 6: Post-reaction solutions performed with CuO/Al2O3 changing the reaction temperature. From the left : 2, 4, 6, 8 hours of reaction at 80°C, 25 bar of oxygen, 500 rpm   The reaction temperature was chosen at 100°C which had less impact on the solution

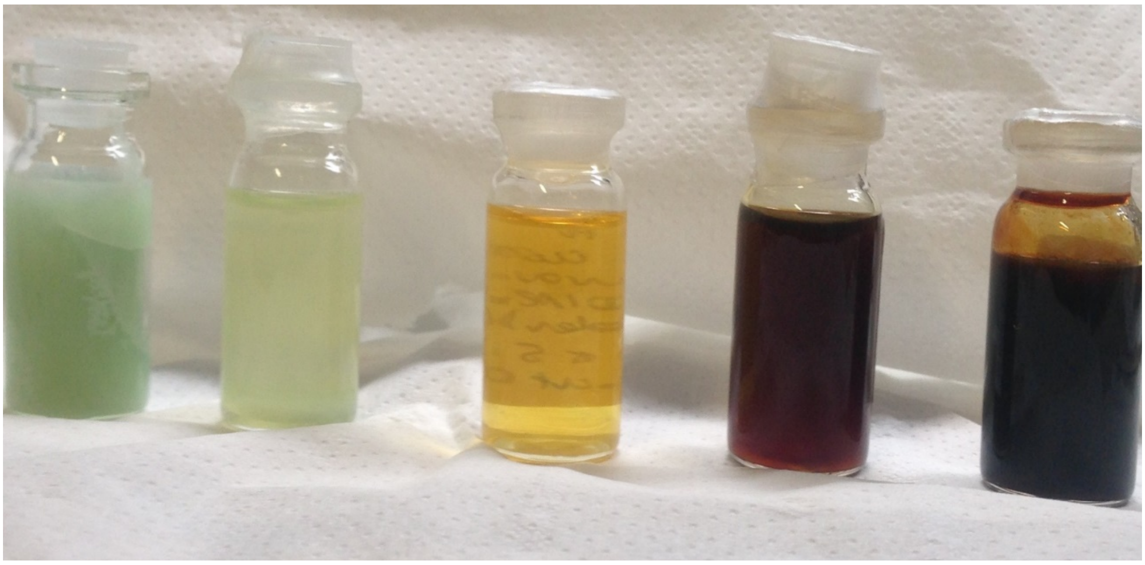

Figure 7: Effect of the reaction time evaluated on the post-reaction mixture using CuO/Al2O3 as catalyst. From the left: :60, 80, 100, 120, 140 °C at 25 bar of oxygen, 5 h and 500 rpm

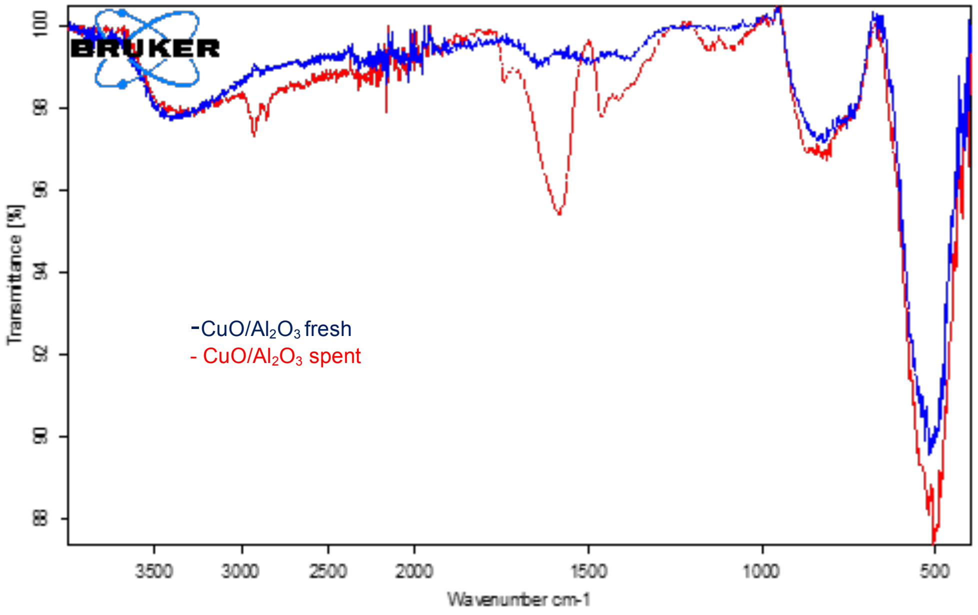

n = 1800 – 1680 cm^{-1} → Carboxylic Acid
n = 1740 – 1660 cm^{-1} → Aldehyde
n = 2900 – 2680 cm^{-1} → Aldehyde
n = 1230 – 1000 cm^{-1} → Alcohol

Figure 8: ATR analysis of fresh (blu line) and spent (red line) CuO/Al_2O_3. The spent catalyst showed the presence of adsorbed organic molecules which are responsible of reduced activity after one cycle of reaction.

Table 2: comparison of different thermal treatments and surface area of the ferrite $Cu_{0,6}Fe_{2,4}O_{4,2}$

SAMPLE	Thermal treatment	$ASS_{B.E.T.(}$ m^2/g)
$Cu_{0,6}Fe_{2,4}O_{4,2}$	Calc. 750°C for 8h (10°C/min)	3
$Cu_{0,6}Fe_{2,4}O_{4,2}$	Calc. 450°C for 8h (10°C/min)	86
$Cu_{0,6}Fe_{2,4}O_{4,2}$	Calc. 250°C for 8h (10°C/min)	231
$Cu_{0,6}Fe_{2,4}O_{4,2}$	Dried 120°C overnight	241

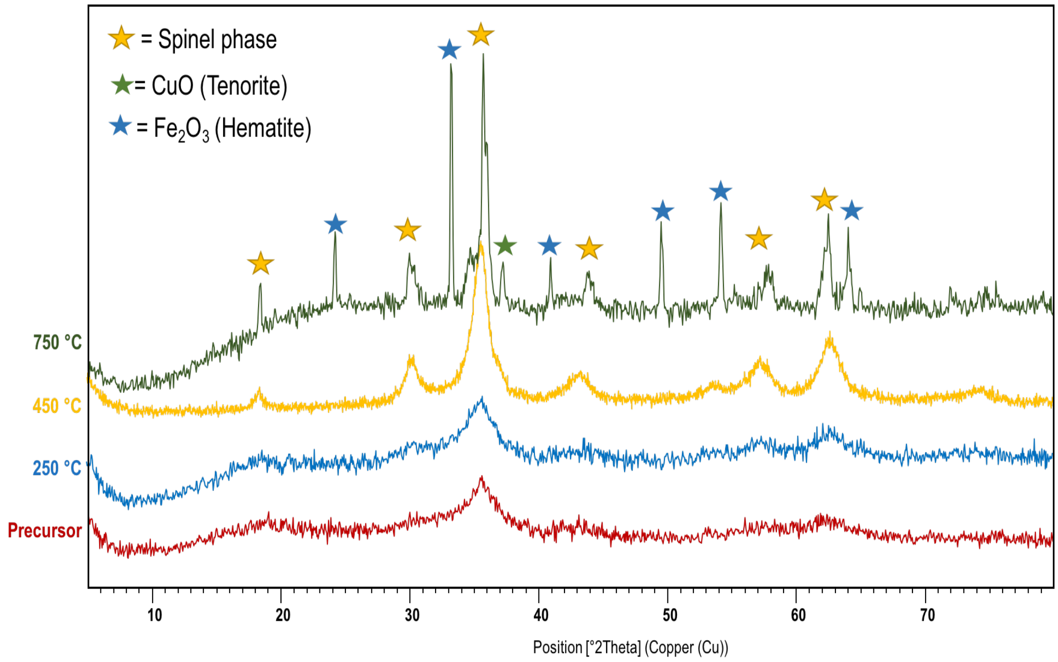

Figure 9: Effect of thermal annealing on the Copper-ferrite catalyst with Cu/Fe ratio 1/4

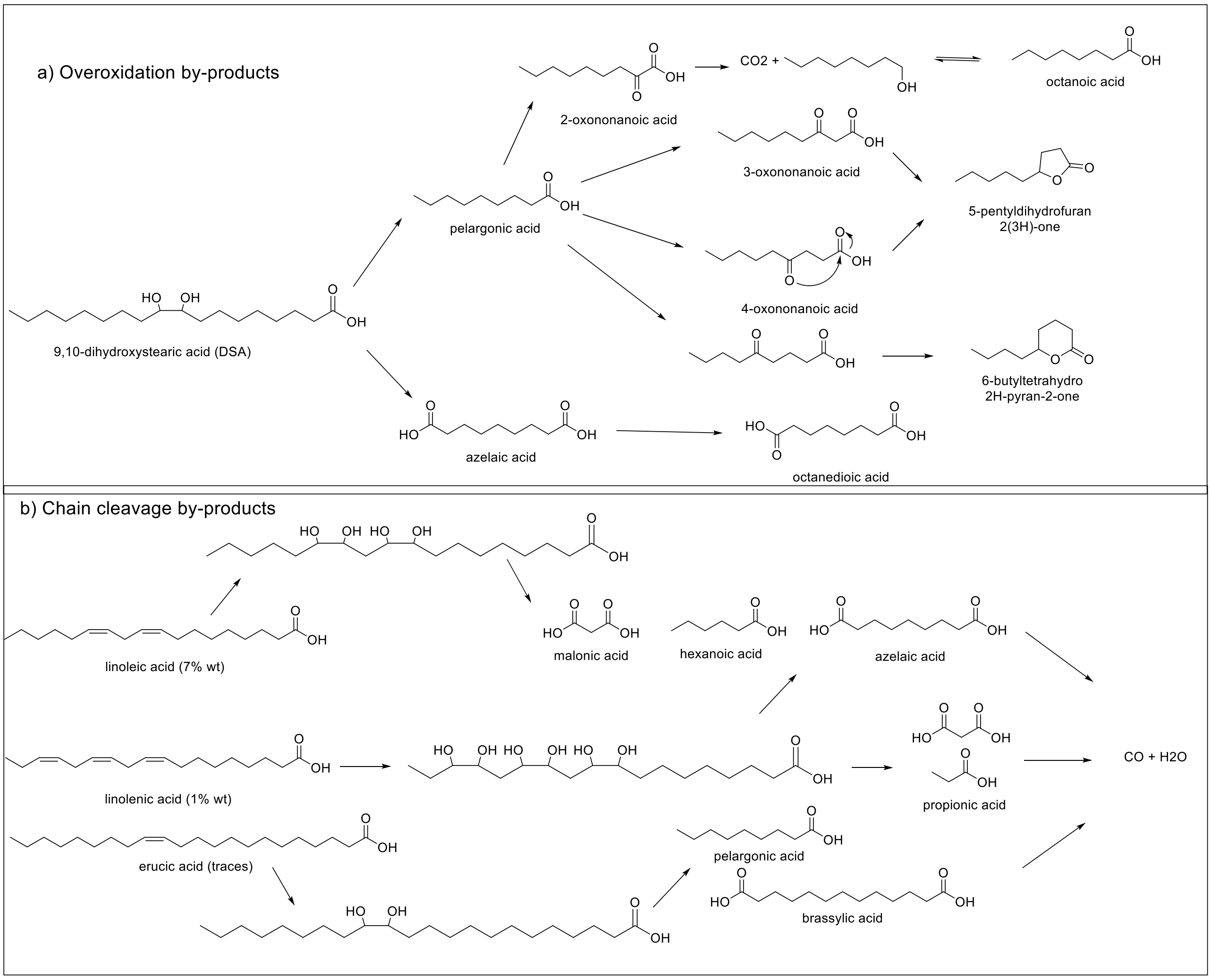

Figure 10: By-products formation scheme due to overoxidation (a) and chain cleavage (b)

Catalysts preparation

Supported Gold nanoparticles. Catalysts were prepared by incipient wetness impregnation of a suspension of gold nanoparticles on titania. The suspension of gold nanoparticles was prepared by synthesis in water according to the modified Turkevich method. Chemicals used were: TiO_2-DT51 (CristalACTiV, > 98 %), Glucose (Sigma-Aldrich, > 99 %), $HAuCl_4$ (Sigma-Aldrich, > 99 %), NaOH (Sigma-Aldrich, >99 %), PVP (Sigma-Aldrich). The synthesis was conducted in water and it consists in the reduction of Au^{3+} by glucose in a basic environment of NaOH using polyvinylpyrrolidoneas stabilising agent for the nanoparticles. The molar ratio used for the synthesis were the following: Au: glucose :NaOH : PVP = 1 : 2 : 8 : 2.75.The final suspension concentration was about $5 \cdot 10^{-3}$ M, with a volume of 100 mL. The synthesis started with the dissolution of the right amount of $HAuCl_4$ in 10 mL of distilled water. PVP and sodium hydroxide were dissolved in 90 mL of distilled water in a three necked round bottom flask provided with magnetic stirrer (600 rpm), thermometer and vapour condenser, and heated up to 95 °C in a glycol bath. When the desired temperature was reached, glucose was added to the solution and the mixture was warmed up for no more than 30 seconds to avoid excessive sugar degradation. Then the gold solution was added and after 2.5 minutes, the reaction was quenched by fast cooling of the flask in ice bath. The final gold suspension showed a dark red colour. The supported catalyst (1,5 wt% Au on TiO_2) was prepared by impregnating the support with the suspension of gold nanoparticles. The precise amount of suspension was weighted and concentrated by centrifugation in tubes fitted with filters of cellulose (50 kDaAmicon Ultra Filters, Millipore) at 1700 rpm for no more than 25 minutes. After the incipient wetness impregnation of the concentrated suspension, the catalyst was dried overnight at 120 °C in order to remove the liquid; then it was washed with boiling water for 30 minutes with the aim of removing the adsorbed PVP, it was dried ad night at 120 °C and finally calcined in static air at 300 °C for 3 hours. The final catalyst colour was a light violet.

Iron-substituted polyoxometalate. A Keggin-type P/W/Fe heteropolyacid with composition $H_4[PW_{11}Fe(H_2O)O_{39}]$was synthesized according to the procedure described by Matveev and co-workers[39]. First, 0.86 g of H_3PO_4 and 2.5 g of $Fe(NO_3)_3$ were dissolved separately in 10 mL and 15 mL of deionized water, respectively. Subsequently, the two solutions were mixed together in a separating funnel and then added drop-by-drop into an aqueous solution of Na_2WO_4 (22.5 g in 25 mL of H_2O), kept under continuous stirring. After 5 minutes from the complete addition of the solution, a beige-yellowish precipitate formed, which was stirred at room temperature for 15 min.

At this point, about 10 mL of an aqueous solution of H_2SO_4 was added dropwise to the mixture and the yellowish solid disappeared. In the acid environment, the formation of $4H^+$ $[PW_{11}Fe(H_2O)O_{39}]^{4-}$ was promoted. After 10-15 min of continuous stirring, a new yellow precipitate was formed. The final precipitate was separated by filtration on a Büchner funnel and dried at 120°C overnight.
